# Shaping the Future of Audiology: Current and Evolving Landscape of Audiology Education

**DOI:** 10.1055/s-0046-1825795

**Published:** 2026-10-06

**Authors:** Jonette Owen, Hannah Siburt, Devon Weist

**Affiliations:** 1 Osborne Audiology Department, College of Nursing and Health Professions, Drexel University, Elkins Park, Pennsylvania, United States; 2 Division of Speech and Hearing Sciences, University of North Carolina at Chapel Hill, Chapel Hill, North Carolina, United States

**Keywords:** Doctor of Audiology (AuD), audiology education, competency alignment, clinical education, interprofessional education

## Abstract

The Doctor of Audiology (AuD) has served as the entry-level degree for more than 25 years, yet audiology education continues to evolve in response to changing healthcare delivery, expanding professional responsibilities, and persistent workforce challenges. This article reviews the historical development of AuD education and examines the current educational landscape through analyses of enrollment trends, degree production, accreditation standards, applicant pathways, curriculum design, and clinical education. Although educational reform alone cannot resolve the profession's broader workforce and economic challenges, the review argues that education remains a critical lever for preparing graduates to practice as autonomous healthcare providers. Key priorities include broadening and diversifying recruitment beyond traditional communication sciences pathways, adopting competency alignment, strengthening clinical reasoning through simulation and standardized assessment, reexamining the structure of the fourth-year externship, and aligning accreditation standards with contemporary practice expectations. The article further emphasizes the importance of professional identity formation and interprofessional education in preparing graduates for collaborative, patient-centered care. Collectively, these recommendations position audiology education as a dynamic and evolving enterprise that must continually adapt to advances in healthcare, workforce demands, and the profession's expanding scope of practice while maintaining educational excellence and public accountability.

## Introduction

The audiology workforce faces compounding structural pressures: constrained workforce growth relative to peer health professions, wage trajectories that have not kept pace with doctoral-level training requirements, rising educational debt that strains early-career financial stability, and persistent gaps between the scope of practice audiologists are trained to deliver and the authority they are legally and economically permitted to exercise. Analyses presented elsewhere in this special issue demonstrate that these challenges are not primarily educational in origin—they are rooted in professional taxonomy, reimbursement architecture, and federal classification structures that have not evolved alongside the Doctor of Audiology (AuD). Educational reform alone cannot resolve them. However, education remains a critical and underutilized lever: the profession cannot claim the authority it seeks if graduates are not consistently and fully prepared to exercise it.

This article examines the current landscape of AuD education with attention to the structural conditions shaping workforce sustainability. Drawing on longitudinal enrollment and degree-production data from the CSD Education Survey, accreditation standards from both the Council on Academic Accreditation (CAA) and the Accreditation Commission for Audiology Education (ACAE), and an analysis of pipeline demographics and educational design, the article identifies persistent gaps between current training models and the competencies required for autonomous clinical practice. Despite more than 25 years since the inception of AuD programs, changes in educational structure, admissions, curriculum, and accreditation have been slow to align with the evolving scope and professional demands of a doctoral-level health profession.

The article advances several interconnected arguments. First, the applicant pipeline must be broadened and diversified beyond traditional communication sciences pathways—but outreach alone is insufficient without concurrent improvements to the profession's economic value proposition. Second, educational models must shift from opportunistic clinical exposure toward competency-based frameworks that produce consistent preparation across programs, including the diagnostic, managerial, and interprofessional competencies required for full-scope autonomous practice. Third, the structure of clinical training—particularly the fourth-year externship—warrants reform to reduce the financial burden that compounds the debt-to-income challenges documented in this issue. Fourth, accreditation standards, as currently structured, establish a minimum threshold but do not consistently drive the educational innovation or scope-aligned preparation the profession requires. Reimagining audiology education within this structural context is not peripheral to the profession's reform agenda—it is the foundation upon which expanded scope, practitioner recognition, and durable workforce sustainability depend.

## History of the Doctor of Audiology

Understanding the pace at which educational change has occurred over time requires first examining how audiology education evolved and the assumptions embedded in its original structure. As demands in technology, science, higher education, and healthcare have continued to evolve, the profession of audiology has now reached more than 25 years since adopting the clinical doctorate as the entry-level practice degree. The transition to the AuD was grounded in the profession's historical development and its response to sustained advances in science and technology, which necessitated an expanded scope of clinical practice.

Audiology emerged as a distinct profession in the mid-20th century, largely in response to the needs of World War II veterans requiring assessment and rehabilitation for hearing disorders associated with prolonged and excessive noise exposure. Delivering these services required interdisciplinary collaboration among specialists in acoustics, speech pathology, psychology, and related fields. 1 Unlike other healthcare professions that were practiced clinically long before becoming institutionalized—such as medicine, dentistry, optometry, and nursing—audiology originated within academic settings and subsequently transitioned into clinical practice. 2

Early audiology training programs were typically embedded within speech pathology curricula rather than existing as independent educational pathways. 3 In 1959, the American Speech-Language-Hearing Association (ASHA) established the American Board of Examiners in Speech Pathology and Audiology and began awarding professional certification in both disciplines. Although this credential did not confer legal authority to practice, it provided a nationally recognized standard of professional competence at a time when most states had not yet implemented licensure systems.

From the 1960s through the 1980s, a master's degree was the standard educational requirement for entry into clinical audiology. Individuals pursuing advanced study or academic careers typically complete a Doctor of Philosophy (PhD). Both the master's and the PhD were academic degrees designed primarily to provide disciplinary knowledge and, in the case of the PhD, research expertise, rather than structured preparation for autonomous clinical practice.

By contrast, professional degrees are designed to prepare individuals for entry into a specific profession and are structured around real-world application. 2 These programs emphasize not only didactic coursework but also extensive supervised clinical training, with curricula aligned to licensure requirements for independent practice. Examples of health professional degrees include medicine (MD), dentistry (DDS or DMD), and optometry (OD).

As audiology matured as a clinical profession, its scope of practice expanded substantially. Advances in diagnostics, amplification technology, vestibular assessment, electrophysiology, and intraoperative monitoring increased both the complexity and responsibility of audiologic care. In healthcare, scope of practice defines the knowledge, skills, and procedures that a licensed professional is legally authorized to perform. While professional organizations such as the American Academy of Audiology (AAA), the Academy of Doctors of Audiology (ADA), and ASHA provide guidance on contemporary competencies, legal scope of practice is determined by state licensure laws and regulated by state licensing boards.

Educational programs play a central role in preparing graduates to meet these evolving expectations, and accreditation standards are designed to align training with competencies required for safe and effective practice. As audiology's scope broadened during the latter half of the 20th century, the limitations of the master's-level training model became increasingly apparent. This growing mismatch between educational preparation and clinical responsibility laid the groundwork for the profession's eventual transition to a clinical doctorate. 2 3 4

Beginning in the late 1970s, leaders within the profession formally examined the need for doctoral-level clinical education. In 1978, ASHA convened a task force to evaluate the necessity of a clinical doctorate in audiology. After several years of deliberation, the task force recommended in 1984 that the profession move toward a professional clinical doctorate. These discussions continued at the 1987 ASHA conference during a panel titled *The Future of Audiology* , where participants reached consensus that audiology should evolve into an autonomous, doctoring profession with limited-licensure practitioner status and direct patient access, transitioning away from master's-level preparation. 4

Parallel discussions also addressed audiology's professional identity and organizational home. Questions emerged regarding whether audiology should remain within a multidisciplinary professional organization alongside speech-language pathology or establish an independent professional structure. Although the Academy of Dispensing Audiologists (founded in 1977) existed as an alternative, it was widely viewed as focused primarily on dispensing and private practice. In January 1988, Dr. James Jerger convened a group of 32 established audiologists to advance these conversations into concrete action, leading to the formation of the AAA. 5 That same year, the Academy of Dispensing Audiologists sponsored an education conference focused on doctoral-level training. 4 In 1991, AAA issued a position paper supporting the development of AuD programs within university settings.

In 1994, the Audiology Foundation of America (AFA) awarded Baylor College of Medicine a grant to establish the first AuD program, marking a critical step in operationalizing the profession's vision for clinical doctoral education. 5 The following year, AFA sponsored the AuD Standards and Equivalency Conference, bringing together representatives from AAA, ADA, and ASHA to develop educational standards for AuD programs. These efforts facilitated the transition from 2-year master's programs to 4-year residential doctoral programs and aligned competency expectations with the expanding scope of audiology practice.

Following these foundational initiatives, AuD programs expanded rapidly. By 1998, six residential AuD programs had been established and accredited by the CAA, the accrediting body for audiology and speech-language pathology programs. Recognizing the need to provide advanced training opportunities for practicing clinicians, Nova Southeastern University launched the first distance-learning AuD program in 1999, enabling master's-level audiologists to complete doctoral training while remaining in practice. 4

From the early 2000s to the present, the number of AuD programs has continued to grow. As of 2026, 77 programs are CAA accredited to grant the AuD degree. 6 In 2003, the ACAE was established by AAA and ADA as a profession-driven accrediting body focused specifically on practice-doctorate preparation in audiology. The creation of ACAE introduced a dual-accreditation environment alongside CAA, offering programs additional pathways to demonstrate alignment with clinical and educational competency standards. To date ACAE has 8 accredited programs and 2 programs in candidacy. 7

The growth and diversification of AuD programs have shaped the contemporary educational landscape, which is characterized by variation in program size, clinical training models, institutional resources, and geographic distribution. These structural differences continue to influence audiology workforce development and professional preparation today. Notably, the proliferation of programs departed from the original vision of the AuD's architects, who anticipated fewer programs with larger enrollments—a consolidation model intended to centralize resources and reduce per-student instructional costs. The current landscape of 77 accredited programs by CAA and 8 accredited programs by ACAE, of which 5 programs are dually accredited and 3 programs hold solo ACAE accreditation, many operating in small cohorts, has instead produced the high per-student costs and limited institutional subsidy structures that contribute to the debt burden examined in examined in the article on "Economic Implications of Audiology Student Debt" in this issue. These developments laid the structural and philosophical groundwork for the debates that would accompany audiology's transition to a clinical doctorate.

## Professional Debates Surrounding the AuD

The transition to the clinical doctorate was accompanied by substantial debate among audiologists, professional associations, accreditation bodies, and other healthcare stakeholders. Concerns centered on several key issues. From an economic perspective, extending the length of training raised questions about increased tuition costs and the potential widening of the gap between graduate debt and entry-level salaries. Educationally, questions emerged regarding how programs would expand curricular content while maintaining instructional quality and adequate clinical training experiences. Some also expressed concern that positioning the clinical doctorate as the preferred entry-level degree could discourage pursuit of PhD training, thereby constraining the profession's research pipeline.

Additional debate arose as advances in cochlear implant programming, vestibular assessment, and other subspecialty areas expanded audiology's scope of practice. Some observers viewed these developments as encroaching on the established roles, competencies, and clinical services of other healthcare professions, raising questions about professional boundaries and interprofessional relationships.

Despite these concerns, proponents of the clinical doctorate articulated clear goals: to enhance clinical training, refine technical competencies, and elevate audiology to a level of professional autonomy comparable to other healthcare fields that had transitioned to clinical doctorates, such as dentistry (DDS/DMD) and optometry (OD). 8 These professions were frequently cited as models in which advanced professional training supported independent clinical decision-making, direct patient management, and, in some cases, diagnostic and therapeutic authority without physician oversight.

Early advocates of the AuD also envisioned structural changes in educational delivery. Specifically, they anticipated fewer training programs with larger enrollments per program, 5 mirroring models seen in other professional doctorates such as physical therapy (DPT) and pharmacy (PharmD). 9 This consolidation was intended to centralize resources, including specialized equipment and faculty expertise, while meeting growing healthcare demands for a larger, highly trained audiology workforce.

Notably, audiology's transition occurred alongside similar shifts in other allied health professions. During the same period, physical therapy, pharmacy, and nursing were also moving toward clinical doctorates as entry-level degrees. Today, clinical doctorate preparation is considered the minimum educational level required for a select group of healthcare professions in the United States. 9 As continued advances in science, technology, and research reshape healthcare intervention and treatment, the need for rigorous, high-quality educational preparation in audiology remains evident.

While the AuD is now firmly established as the entry-level degree for clinical audiology practice in the United States, we contend that maintaining the status quo in educational standards risks limiting the profession's future growth. Windmill 10 discusses a reactive–proactive framework in the context of hearing healthcare delivery, highlighting how professions that respond only after external pressures arise risk stagnation. Although their work focused on service delivery, this framework is equally applicable to audiology education and training.

If the profession relies primarily on accrediting bodies, legislative action, or market forces to drive educational change, audiology risks allowing its evolution to be shaped externally rather than intentionally directed from within. By contrast, articulating a proactive, internally driven vision—one that is forward-looking, responsive to emerging clinical demands, and informed by successful educational models across healthcare—offers an opportunity to shape the next phase of professional development deliberately. In doing so, the profession honors the original goals of the AuD movement, which emphasized professional autonomy and positioned audiologists as leaders in hearing and balance healthcare.

These debates regarding autonomy and scope were not purely philosophical; they materially influenced how AuD programs were structured, accredited, and positioned within academic institutions.

## Academic Placement of AuD Programs

AuD programs are situated within a wide range of academic structures across universities and colleges. Historically, these programs have been housed in departments or schools of communication sciences and disorders (CSD), audiology, health sciences, or medicine. This variation reflects both audiology's origins within speech-language pathology and its continued evolution toward a broader role within healthcare.

The academic home of an AuD program is more than an administrative designation; it carries meaningful implications for educational philosophy, resource allocation, clinical training opportunities, and professional identity formation. Where programs are positioned within an institution influences not only the learning environment but also how audiology is perceived by students, faculty, and other health professions—both within academia and in the communities where graduates ultimately practice.

Programs housed within colleges of health sciences or medical schools may provide access to interprofessional education (IPE), shared simulation laboratories, integrated clinical rotations, and exposure to team-based models of care alongside other healthcare professions. In contrast, programs located within CSD units—and in liberal arts-related colleges—may place greater emphasis on rehabilitative, behavioral, and counseling-oriented models rooted in speech-language pathology traditions. These differing academic homes—summarized in Table 1 —contribute to variation in clinical exposure, training models, and the development of professional identity among AuD students.

**Table 1:** Administrative location of communication sciences and disorders (CSD) within U.S. higher education institutions 23

This structural variability across institutions highlights a broader challenge in audiology education: the absence of a fully unified framework for preparing audiologists as autonomous healthcare professionals across diverse practice settings. 11 Understanding the impact of academic placement is, therefore, an important step in evaluating how current educational structures align—or fail to align—with the competencies required in contemporary healthcare environments. The stakes extend beyond training quality: graduates who emerge from medically integrated environments with strong diagnostic identities and interprofessional credibility are better positioned to claim the full clinical authority that scope modernization and Medicare practitioner recognition require. Academic placement, in this sense, is not merely an institutional decision—it is a professional positioning strategy with downstream implications for workforce utilization (see "Audiology at the Intersection of Labor, Revenue, and Professional Taxonomy"), scope of practice recognition (see "Audiology's Nomenclature and Taxonomy Under the Law"), and the economic authority (see "Professional Status of Audiology in U.S. Healthcare") examined elsewhere in this issue.

### Core Educational Components of AuD Programs

Regardless of academic placement, the overarching goal of all AuD programs is to provide a comprehensive curriculum that prepares graduates for independent clinical practice. 2 Audiologists are trained to evaluate, diagnose, treat, and manage hearing and balance disorders across the lifespan. Core components of AuD education typically include:

- Didactic coursework: coursework encompasses a broad range of topics related to audiology, hearing science, and human health. Common areas include anatomy and physiology of the auditory and vestibular systems, hearing science, psychoacoustics, speech and language development, diagnostic audiology, rehabilitative techniques, amplification and assistive listening technologies, hearing conservation, counseling, professional ethics, and healthcare systems.
- Clinical training: supervised clinical training is central to AuD education. Students complete hands-on clinical rotations under the supervision of licensed audiologists serving as clinical preceptors. Training occurs across a variety of settings, including hospitals, clinics, private practices, schools, and rehabilitation centers, and involves patients ranging from infancy through older adulthood across diverse socioeconomic contexts.
- Research exposure: research opportunities enhance students' understanding of evidence-based practice and support the development of critical thinking skills. While the depth of research engagement varies by program, exposure to research methods and scientific inquiry remains an important component of doctoral-level preparation.
- IPE: interprofessional education allows students to experience the collaborative nature of healthcare delivery. Opportunities to engage with professionals from related disciplines promote a more holistic approach to the management of hearing and balance disorders.

### Professional Development

Professional development training includes clinical communication skills, cultural competence, ethical decision-making, professional responsibilities, and reflective practice, all of which are essential for effective and sustainable clinical careers. 12

Institutional context and educational variability: the academic home of an AuD program can shape how these core components are delivered. Programs housed within CSD units often emphasize behavioral and rehabilitative models grounded in the speech and hearing sciences. Those located in colleges of health sciences may benefit from interprofessional coursework, shared clinical resources, and collaboration with professions such as physical therapy, occupational therapy, nursing, and physician assistant studies. Programs affiliated with medical schools or departments of otolaryngology frequently provide immersive exposure to medical environments, advanced diagnostic technologies, surgical collaboration, and biomedical research infrastructure.

This institutional diversity offers both opportunities and challenges. While varied academic homes can enrich the profession by exposing students to different perspectives and practice models, they also contribute to inconsistencies in the scope, depth, and uniformity of clinical preparation across programs. These differences underscore the importance of ongoing evaluation of educational standards to ensure that all graduates—regardless of program structure—enter the workforce with competencies aligned to current and emerging healthcare demands.

Data from the 2024-2025 CSD Education Survey, 23 summarized in Table 1 , illustrate the current administrative placement of CSD programs nationwide. Based on self-reported data, approximately 60% of programs are housed within units categorized as “Allied Health Sciences,” “Health Professions,” or “Public Health,” highlighting the profession's increasing integration within broader healthcare academic structures. These differences in academic home are not accidental; they are enabled—and constrained—by accreditation structures that permit wide variation in program design.

## Role of Accreditation in Program Placement

Accreditation standards for audiology education do not prescribe a specific academic home for AuD programs beyond general institutional accreditation requirements. A foundational limitation must be acknowledged at the outset: accreditation establishes a minimum compliance threshold, not a marker of educational excellence or alignment with the full scope of practice the profession is seeking to claim. A program may satisfy all required criteria—coursework coverage, faculty qualifications, clinical hours—while still producing graduates with uneven preparation in diagnostic reasoning, competencies associated with expanded scope, and the professional identity necessary for autonomous practice. This gap between compliance and excellence is not incidental; it is a structural feature of the current accreditation framework that has contributed to the inconsistencies in workforce readiness documented elsewhere in this issue. Regardless of where programs are housed within a university, all accredited AuD programs must meet the standards established by either the CAA in Audiology and Speech-Language Pathology 6 or the ACAE. 7

### Accreditation in Higher Education

Accreditation has long served as a cornerstone of quality assurance in higher education. It is a formal process through which colleges, universities, and academic programs are evaluated to ensure they meet minimum standards related to curriculum, faculty qualifications, student support services, assessment, and overall institutional effectiveness. The primary purpose of accreditation is to promote educational quality and to ensure that programs adequately prepare students for academic achievement and professional practice.

Although accreditation exists at both the institutional and programmatic levels, the focus of this discussion is programmatic accreditation—the discipline-specific process used to evaluate professional education programs. Programmatic accreditation assesses whether a given program meets established standards related to curriculum, faculty qualifications, clinical training, student outcomes, and program resources. 6 7 The process for initial accreditation or reaccreditation is generally similar across accrediting bodies and involves a comprehensive self-study aligned with accreditor standards, followed by a site visit from peer reviewers—typically faculty members and licensed professionals—who assess compliance and gather additional evidence. Accreditation decisions are made by a governing board and may include full accreditation, conditional accreditation with required follow-up, probationary status, or denial or withdrawal of accreditation.

### Audiology-Specific Accreditation Pathways

AuD programs may pursue programmatic accreditation through either the CAA in Audiology and Speech-Language Pathology or the ACAE. For many decades, CAA served as the sole accrediting body for both audiology and speech-language pathology programs, reflecting the professions' shared historical roots within CSD. CAA continues to accredit graduate programs in both disciplines. The organizational structure and scope of each accrediting body are summarized in Table 2 .

**Table 2:** Organizational structure and scope between the Council on Academic Accreditation in Audiology and Speech-Language Pathology (CAA) 6 and the Accreditation Commission for Audiology Education (ACAE) 7

	Council on Academic Accreditation (CAA)	Accreditation Commission for Audiology Education (ACAE)
Founded	1959	2003
Affiliation	American Speech-Language-Hearing Association (ASHA)	American Academy of Audiology (AAA)
Mission	Assure quality and promote excellence through accreditation of education programs for entry-level audiologists and speech-language pathologists.	Serve the public by establishing, maintaining, and applying standards to ensure the academic quality and continuous improvement of audiology education that reflects the evolving practice of audiology.
Accreditation recognition	−Council for Higher Education Accreditation and the Secretary of the U.S. Department of Education −Member of the Association of Specialized and Professional Accreditors	Council for Higher Education Accreditation and the Secretary of the U.S. Department of Education
Number of audiology programs accredited by each organization a	−77 accredited programs −2 candidate programs	−8 accredited programs −2 developing status
Accreditation cycle	5 or 8 y	10 y
Competencies	Broader communication disorders focus	Audiology-specific focus

a Five programs have dual accreditation.

ACAE was established in 2003 as a profession-centered accrediting body focused exclusively on audiology. Its creation reflected an effort to align accreditation standards more directly with the competencies and clinical expectations of a modern practice doctorate. According to ACAE, its mission is to focus “solely on academic quality and continuous improvement of audiology programs.” 7

Despite differences in organizational structure and affiliation, both accrediting bodies emphasize similar core domains, including administrative organization, faculty qualifications, curriculum (didactic and clinical), student assessment, and program resources. Both also require outcome measures designed to evaluate program effectiveness and student competency.

The availability of two accreditation pathways provides programs flexibility in aligning accreditation with institutional mission and educational philosophy while maintaining shared expectations for educational quality. At the same time, accreditation standards do not prescribe academic placement within a university, allowing substantial variability in where AuD programs reside and contributing to the diversity of educational models across the profession.

Accreditation standards establish baseline requirements for programmatic quality. They verify that programs provide evidence of preparing graduates for doctoral-level entry into clinical practice. However, state licensure laws—rather than accrediting bodies—ultimately define the legal scope of practice. As scope evolves, accreditation standards must periodically be reassessed to ensure alignment with contemporary and future clinical expectations.

Importantly, accreditation represents a minimum threshold rather than a marker of educational excellence. 6 7 A program may meet required criteria related to coursework, faculty qualifications, and clinical hours yet still vary in how effectively it develops advanced diagnostic reasoning, clinical judgment, and professional accountability. Accreditation ensures compliance; it does not inherently drive innovation or continuous improvement. Responsibility for advancing educational quality therefore rests not only with accreditors, but also with individual programs and the profession as a whole.

A related consideration involves the structural and financial implications of the two accrediting pathways. Because most AuD programs are housed within units that also include speech-language pathology, CAA accreditation is often administratively efficient for universities, with accreditation costs bundled across programs. 6 In contrast, ACAE accreditation applies exclusively to audiology and requires separate institutional commitment. 7

In addition to differing financial costs, Table 2 displays that CAA accreditation reflects standards developed for both audiology and speech-language pathology, consistent with their shared historical roots within CSD. ACAE, by contrast, was established with the explicit mission of supporting audiology-specific competencies and the preparation of audiologists as autonomous healthcare professionals.

As of 2026, approximately 77 AuD programs are accredited by CAA, 8 by ACAE, and 5 hold dual accreditation, with additional programs pursuing a second pathway ( Table 2 ). Given the relatively small number of AuD programs nationally, the presence of two accrediting bodies raises questions about resource allocation and long-term sustainability. At the same time, considerations of efficiency must be balanced with longstanding professional goals related to autonomy, competency development, and leadership in hearing and balance healthcare.

Taken together, these issues highlight a broader challenge: reliance on accreditation standards alone may reinforce a reactive model of educational oversight. Preparing audiologists for evolving healthcare demands requires intentional efforts that extend beyond compliance with minimum criteria. As the profession evaluates its educational infrastructure, it is appropriate to consider whether current accreditation structures remain fully aligned with the vision and competencies required for contemporary and future practice.

## Student as the Entry Point to Workforce Sustainability

While accreditation and institutional structures shape how programs operate, their impact is ultimately realized through the students who enter, progress through, and exit AuD programs. Historically, applicants to AuD programs have entered the profession through undergraduate programs in CSD or through personal experiences with individuals affected by hearing or balance disorders. For many students, audiology emerges as a career option only after exposure to an engaging instructor or a compelling clinical topic—often late in their undergraduate training. While this pathway has served the profession for decades, it has largely functioned as a passive recruitment model, dependent on chance exposure rather than intentional outreach.

At a time when demand for audiologic services continues to increase, reliance on this narrow pipeline constrains the profession's ability to grow and diversify its workforce. Sustaining and strengthening the audiology workforce will require broadening recruitment efforts beyond traditional CSD programs to include students from related healthcare disciplines, biomedical and life sciences, engineering and technology-focused programs, and nontraditional educational pathways.

Trends across higher education further underscore the urgency of this shift. Although overall enrollment patterns continue to fluctuate due to economic and societal factors, the profile of today's undergraduate student has changed in durable ways. 14 Students are increasingly older, more likely to balance employment and family responsibilities, and more likely to enter higher education through indirect or nontraditional routes. These demographic shifts extend into professional degree programs, including audiology, and call for educational models that are responsive to the realities of contemporary learners.

Recognizing these changes reframes the challenge facing audiology education. Rather than viewing stagnant or declining applicant pools as unavoidable, the profession has an opportunity to reconsider how it attracts, prepares, and retains future audiologists. Reimagining audiology education must therefore begin with the student—not only as a learner, but as a future clinician positioned to meet evolving public health needs.

Within higher education more broadly, institutions have increasingly acknowledged students as central stakeholders in the educational enterprise. As a result, many universities have shifted from asking, “How prepared is the student to succeed in this program?” to “How prepared is the institution to meet the broader needs of today's students?” 15 Audiology education is not immune to these shifts. Addressing workforce sustainability will require programs to critically examine recruitment strategies, educational structures, and student support systems. Reimagining the future of audiology must therefore include the student applicant as an essential starting point. ( Figs. 1 and 2 )

**Fig. 1:**
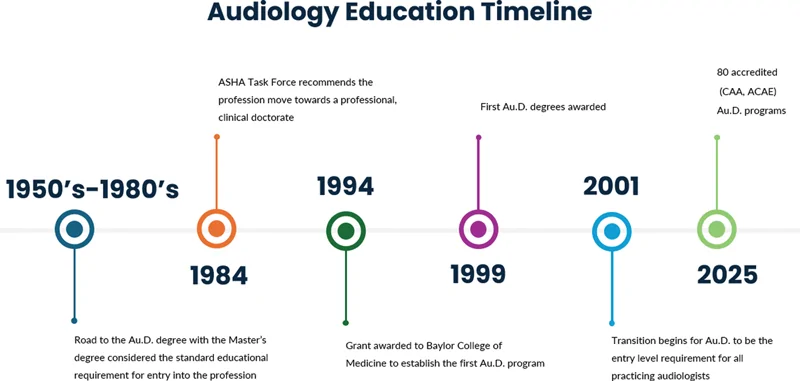
Audiology education timeline.

**Fig. 2:**
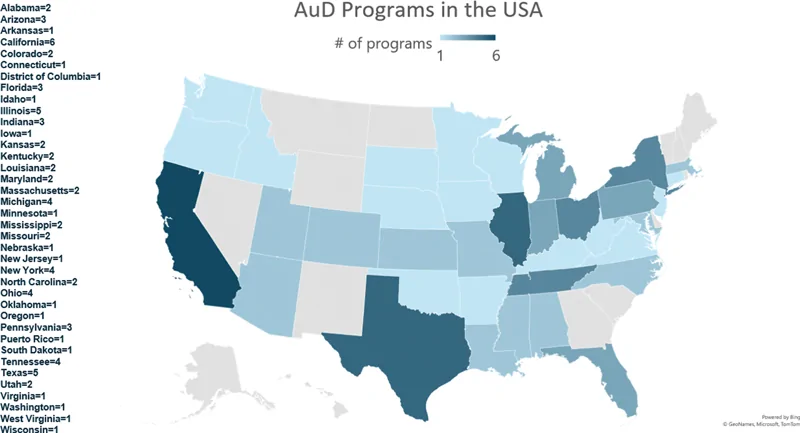
Number of AuD programs by state in the United States. 6 7 (Note: Does not include the AuD program in Puerto Rico.)

## Broadening the Pipeline and Reframing Professional Identity

### Pipeline Demographics

Since 2010, overall enrollment in U.S. higher education institutions has experienced an overall decline, 24 as shown in Fig. 3 . Additionally, data from the National Student Clearinghouse Research Center 14 indicate that between spring 2020 and spring 2021, on-campus enrollment decreased by 4.2%, while enrollment in online programs increased, stemming from the pandemic. More recently, undergraduate on-campus enrollment has shown modest recovery, with a 2.1% increase since the onset of the COVID-19 pandemic.

**Fig. 3:**
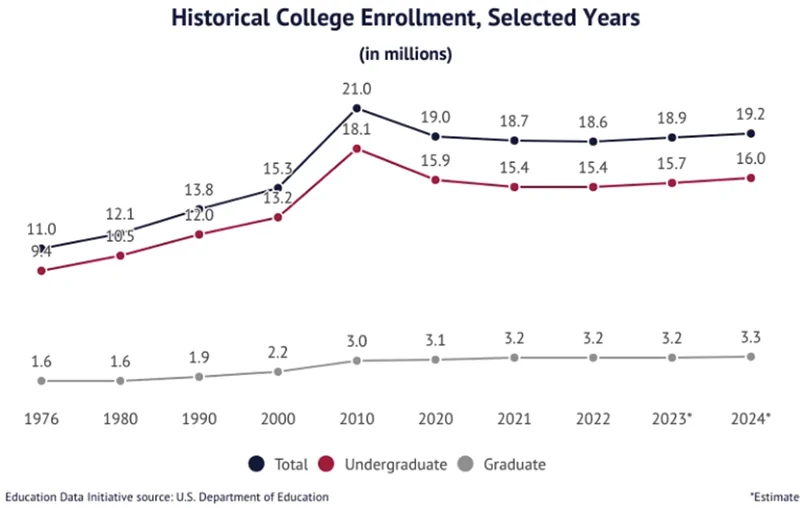
This figure from College Enrollment Statistics (2024) shows historical college enrollment for undergraduate and graduate programs from 1976 to 2022. 24

Community colleges, in particular, demonstrated notable growth, with a 4.4% increase in fall enrollment as of September 2023. This growth has been driven largely by an increase in Black, Latino, and Asian student enrollment, while enrollment among White students has continued to decline at both the undergraduate (−0.9%) and graduate (−1.9%) levels, most sharply among first-year undergraduates (−9.4%).

### CSD and AuD Enrollment Demographics

With respect to enrollment in CSD education (CAA accreditation), data 6 are shown in Figs. 4 to 7 . Fig. 4 illustrates relatively stable enrollment trends in CSD undergraduate programs from 2010 to 2011 to 2023 to 2024, with modest fluctuations over time. Enrollment increased steadily from 9,932 in 2010 to 2011 to a peak of 12,182 in 2021 to 2022, reflecting gradual growth over approximately a decade. A slight dip occurred from 2017 to 2018, followed by renewed growth from 2021 to 2022.

**Fig. 4:**
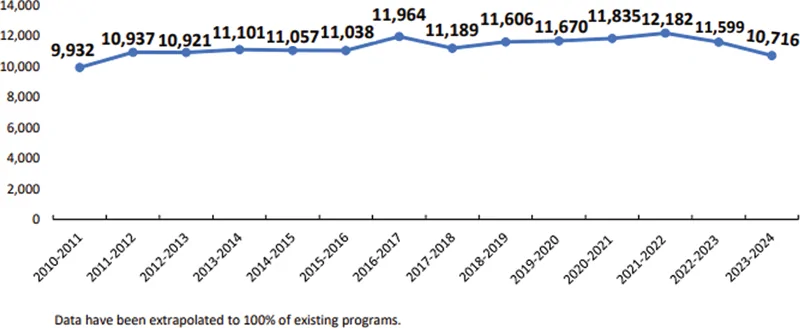
This figure from the CSD Education Survey shows the number of undergraduate degrees granted between 2010 and 2024. 13

**Fig. 5:**
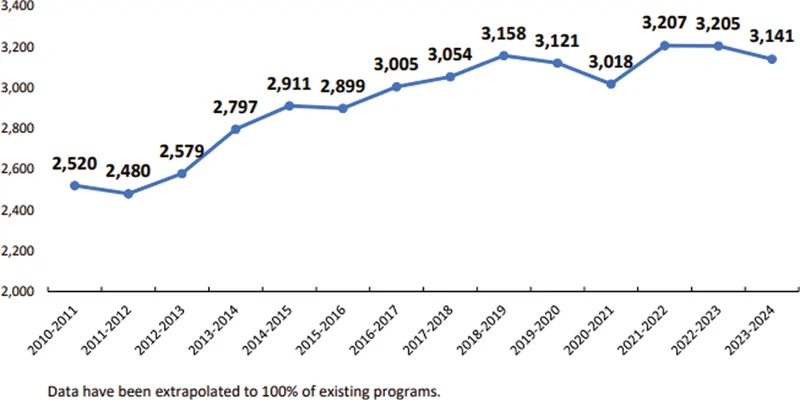
Cumulative number of students enrolled in Doctor of Audiology (AuD) clinical doctorate programs annually between 2010 and 2024. 13

**Fig. 6:**
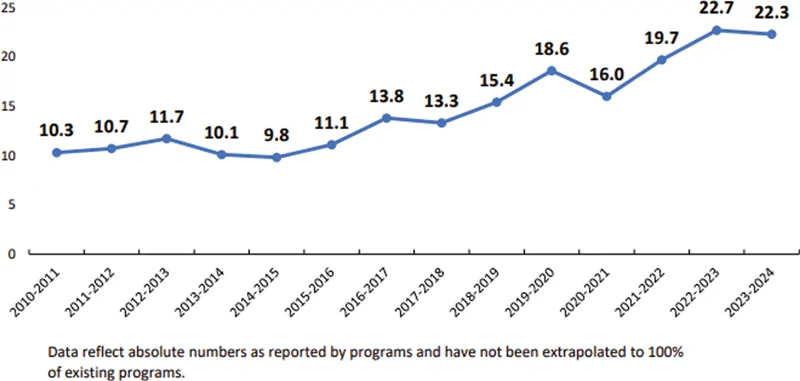
This figure from the CSD Education Survey shows the percentage of racial/ethnic minority students enrolled in audiology clinical doctorate entry-level programs between 2010 and 2024. 13

**Fig. 7:**
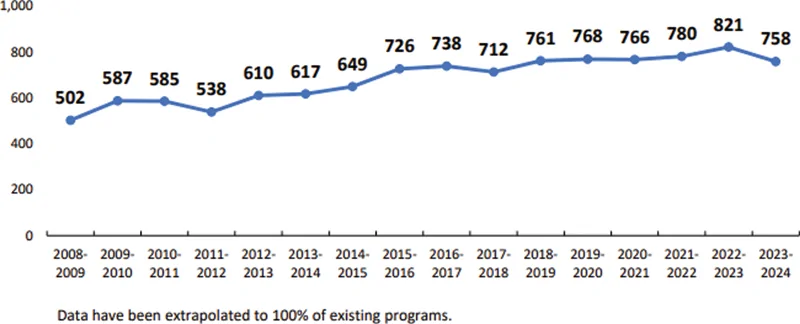
This figure from the CSD Education Survey shows the number of AuD degrees granted between 2008 and 2024. 13

However, the most recent 2 years show a notable decline, dropping from 11,599 in 2022 to 2023 and further to 10,716 in 2023 to 2024. Overall, the trend suggests long-term stability with incremental growth through the early 2020s, followed by a recent contraction that may warrant closer examination in the context of workforce sustainability and undergraduate pipeline dynamics.

Fig. 5 illustrates a steady overall increase in AuD enrollment from 2010 to 2011 through 2018 to 2019, rising from approximately 2,520 to 3,158 students. Following this peak, there is a modest decline from 2019 to 2021, likely reflecting pandemic-era disruptions, before rebounding from 2021 to 2022 to the highest level observed (3,207). Since that peak, growth has slightly tapered but remains elevated relative to the early 2010s, with 3,141 reported in 2023 and 2024.

Overall, the longitudinal trend reflects long-term expansion of AuD degree production over the past decade, punctuated by short-term fluctuations, and suggests relative stabilization in recent years at levels significantly higher than those seen at the beginning of the period.

The data in Fig. 6 indicates a meaningful and sustained increase in racial/ethnic diversity within AuD programs over time, with representation more than doubling since the early 2010s, despite short-term fluctuations. From 2010 to 2011, through approximately 2014 to 2015, representation remained relatively stable, fluctuating between approximately 9.8 and 11.7%. Beginning in 2015 to 2016, the percentage began a sustained upward trajectory, increasing to 13.8% by 2016 to 2017 and continuing to rise through 2019 to 2020 (18.6%).

Although there is a decline in enrollment from 2020 to 2021 (16.0%), likely reflecting pandemic-related disruptions, the trend quickly rebounds. Enrollment reaches its highest levels in 2022 to 2023 (22.7%) and remains elevated in 2023 to 2024 (22.3%). This growth is an encouraging indicator of progress toward a more inclusive and sustainable workforce. Continued diversification strengthens cultural responsiveness, expands service reach, and supports long-term professional resilience.

Finally, Fig. 7 demonstrates a clear long-term upward trend in the number of AuD degrees granted between 2008 to 2009 and 2022 to 2023, followed by a modest decline in 2023 to 2024. From 2008 to 2009, 502 degrees were awarded. Degree production increased steadily over the next several years, surpassing 700 by 2015 to 2016 and continuing gradual growth through 2018 to 2020. After a brief plateau during the 2019 to 2021 period, conferrals rose again, reaching a peak of 821 degrees in 2022 to 2023. The most recent data (2023–2024) show 758 degrees awarded, a slight decline representing a notable change, but not an unprecedented decrease compared with the prior peak. Overall, the trend reflects substantial growth in AuD degree production over the past decade and a half, with periodic fluctuations likely influenced by cohort size variability, enrollment shifts, and broader higher education dynamics.

Taken together, these data in Figs. 4 to 7 suggest that while the profession has expanded degree production over time, recent fluctuations reinforce the need for proactive pipeline and educational strategies.

### Opportunities to Increase the Enrollment Pipeline

As discussed in the workforce supply article in this issue, the number of audiologists entering the profession is insufficient to meet the growth seen in the aging U.S. population. How, then, does audiology broaden and continue to diversify its applicant pool? Expanding the applicant pool requires more than increased program visibility; it necessitates a clear and compelling professional identity. Wilson 25 describes professional identity formation as a process through which individuals internalize the values, norms, skills, and expectations of a professional community, shaped by personal experience, cultural context, and a sense of belonging. Importantly, this process begins well before entry into graduate education and continues throughout a clinician's career.

One potential entry point for strengthening professional identity development is earlier self-assessment and career alignment. Tools—such as the Myers–Briggs Type Indicator 16 or Keirsey Temperament Sorter 17 —are widely available and easily administered, yet are infrequently used to help prospective students explore healthcare career fit. While such assessments are not determinative, they can support individuals in identifying work environments, interpersonal dynamics, and professional roles that align with their preferences and strengths. Greater self-awareness can also enhance interpersonal understanding, a skill that is particularly relevant in patient-centered professions such as audiology.

Early exposure to audiology represents another critical strategy for pipeline development. Outreach efforts targeting middle and high school students, guidance counselors, and educators—particularly those engaging students interested in healthcare, science, music, or technology—can help position audiology as both clinically rigorous and technologically advanced. Parallel engagement with community colleges, technical programs, and allied health pathways offers additional opportunities to diversify and strengthen the future workforce.

However, outreach strategies must be understood within the economic context documented in this special issue. Analyses of audiology wages, debt-to-income ratios, and early-career financial strain (see "Economics of Audiology Wages" and "Economic Implications of Audiology Student Debt for Workforce Sustainability" in this issue) indicate that pipeline constraints are not solely a problem of visibility or awareness. Prospective students who are aware of audiology and who evaluate its economic value proposition—comparing debt burden, early-career wages, and career flexibility against peer health professions—are making rational decisions when they choose alternative pathways.

The evidence reviewed in this issue is consistent with outreach that recruits students into conditions producing financial strain and early attrition, shifting rather than solving the pipeline problem. Meaningful pipeline strengthening therefore requires parallel progress on the structural conditions that make audiology an economically viable and professionally rewarding career: improving wage trajectories, reforming the financing of clinical training, and ensuring that the scope and authority graduates are prepared to exercise are recognized by the systems in which they practice.

Professional identity formation is further reinforced during graduate training. AuD programs housed within health sciences or medical school environments may offer increased opportunities for IPE and collaborative clinical experiences. Such exposure can normalize audiologists' roles as diagnostic and management-oriented clinicians within healthcare teams, contributing to workforce readiness by fostering professional confidence, clinical reasoning, and a stronger sense of legitimacy within the broader healthcare system.

## Educational Models for Workforce Readiness and Consistent Competency

As the profile of audiology students continues to evolve, educational models must evolve in parallel. Contemporary AuD cohorts increasingly include a mix of traditional students entering directly from undergraduate programs and nontraditional learners who bring prior academic or professional experience. Within and across these groups, learning styles, prior preparation, and clinical readiness vary widely.

Reliance on traditional clinical education models—particularly those driven by variable, real-world patient caseloads—is nonlinear and unpredictable, contributing to well-documented inconsistencies in clinical skill development across programs. As reported by CAA, 6 when learning opportunities depend primarily on chance exposure rather than intentional design (e.g., simulations), variability in competency acquisition and clinical aptitude is an expected outcome. Addressing this structural gap requires a shift from a predominantly reactive model of clinical education to a proactive, competency-based approach in which learning experiences are structured, measurable, and aligned with defined outcomes.

Simulation-based education, standardized patient (SP) encounters, and structured clinical problem-solving experiences provide repeatable and equitable learning opportunities that reduce reliance on variable clinical volume. These approaches align audiology education with best practices widely adopted across other health professions. When deliberately designed and systematically implemented, such methods strengthen diagnostic reasoning, clinical communication, and readiness for independent practice while enabling more consistent assessment of student competencies across programs.

### Innovative Clinical Education

If workforce sustainability begins with who enters the profession, it is secured by how consistently and intentionally those individuals are trained. The changing profile of today's audiology student, coupled with growing clinical and public health demands, necessitates a fundamental re-examination of how audiologists are prepared for practice. While recognizing shifts in student demographics is important, meaningful progress depends on how academic institutions respond—specifically, by intentionally redesigning educational models to support workforce readiness, competency development, and professional identity formation.

Across higher education, the overarching goal remains consistent: to prepare individuals for successful and fulfilling professional lives by equipping them with the knowledge, skills, and critical thinking abilities needed to function in increasingly complex healthcare environments. For audiology, this goal extends beyond technical proficiency to include diagnostic reasoning, clinical judgment, effective communication, and interprofessional collaboration. Achieving these outcomes requires moving beyond traditional instructional approaches toward educational models that better reflect how contemporary learners acquire, integrate, and apply knowledge in clinical practice.

### Aligning Audiology Education with Adult Learning Principles

Audiology programs educate increasingly diverse cohorts that include students transitioning directly from undergraduate education as well as nontraditional learners entering from other professional backgrounds. Andragogical theory provides a useful framework for addressing this diversity in learner experience and expectation. Unlike pedagogical approaches, which are primarily teacher-centered and content-driven, andragogy emphasizes self-directed learning, relevance, readiness, and internal motivation—principles well aligned with graduate-level professional education. 26

The six assumptions articulated by Knowles 18 —self-directedness, need to know, readiness to learn, orientation to learning, internal motivation, and reliance on lived experience—closely mirror the expectations placed on audiology students as emerging healthcare professionals. However, assuming that these attributes will develop organically without intentional curricular design risks perpetuating variability in clinical competence and preparedness. Bridging the gap between traditional educational structures and the needs of adult learners therefore requires purposeful instructional strategies that foster learner autonomy while ensuring consistent, measurable outcomes across programs.

### From Variable Clinical Exposure to Competency-Based Preparation

One of the most persistent challenges in audiology education is the reliance on opportunistic clinical exposure to develop essential clinical skills. While clinical rotations are indispensable, they are inherently variable, shaped by patient availability, case mix, and site-specific practice patterns. Consequently, students may graduate with uneven preparation in diagnostic reasoning, patient communication, and competencies across the full scope of practice.

To address these inconsistencies, audiology education must shift from a reactive model—one that depends largely on clinical happenstance—to a proactive, competency-based approach. Integrating structured clinical problem-solving, case-based learning, and simulation as core components of the curriculum provides a mechanism for ensuring repeatable, equitable learning experiences. These approaches enable educators to deliberately target competencies aligned with workforce expectations while affording students multiple opportunities to practice, reflect, and refine their clinical skills.

Clinical problem-solving and simulation-based education have been widely adopted across other health professions to strengthen diagnostic reasoning, test selection, synthesis of clinical information, and development of differential diagnoses. Although such strategies have been implemented within audiology programs to varying degrees, the absence of consistent expectations across programs has limited their collective impact. Establishing clearer benchmarks for simulation and case-based learning within accreditation standards could promote greater national consistency and better prepare graduates for independent clinical practice.

### Simulation and Standardized Patients as Core Educational Tools

Simulation-based education gained prominence in audiology during the COVID-19 pandemic, when educators were compelled to identify alternative approaches to clinical instruction. Although many of these strategies emerged out of necessity, their continued use underscores the value of simulation as a permanent and intentional component of audiology education. Simulation offers a controlled learning environment in which students can engage in deliberate practice without the time pressures, productivity demands, or patient-safety constraints inherent to live clinical care.

SPs, long established in medical education, provide a structured and evidence-based method for developing both technical and interpersonal competencies. SPs are trained to present scripted medical histories, symptoms, and psychosocial contexts, allowing students to practice clinical encounters while receiving targeted feedback related to communication, professionalism, and clinical reasoning. Incorporating SP encounters into audiology curricula supports earlier and more consistent development of skills that are often deferred until later stages of clinical training.

Building on this model, the use of nontraditional standardized patients (N-SPs)—individuals with authentic diagnoses and lived experiences rather than trained actors—offers additional educational value. N-SP encounters require students to integrate case history, diagnostic testing, differential diagnosis, and results counseling within a structured, lower-stakes environment. Unlike traditional clinical rotations, where students may primarily perform testing under supervision, N-SP sessions allow learners to assume greater responsibility for patient interaction, clinical interpretation, and care planning earlier in their training.

Equally important is the structured debriefing that follows SP and N-SP encounters. Guided reflection, performance feedback, and discussion of communication strategies reinforce learning, promote self-awareness, and support professional identity formation. Enhancements such as video review and patient feedback instruments further strengthen these experiences and provide objective data for competency assessment and program-level evaluation.

### Integrating Interprofessional and Innovative Learning Strategies

These same simulation and problem-solving frameworks also provide a natural entry point for meaningful IPE. Audiology does not exist in isolation within the healthcare system, yet it remains underrepresented in many IPE initiatives. Expanding simulation- and case-based learning to include interprofessional contexts—such as medical, nursing, and rehabilitation training environments—positions audiology as an integral contributor to comprehensive patient care. Updating existing interprofessional cases to explicitly incorporate hearing and balance considerations enhances clinical realism and reinforces audiologists' roles within diagnostic and treatment teams.

Innovative instructional strategies adopted in other health professions also offer valuable models for audiology education. Educational escape rooms, for example, promote collaboration, critical thinking, and problem-solving within goal-oriented learning environments. When aligned with clearly defined educational objectives, these approaches increase learner engagement, reinforce teamwork, and strengthen knowledge retention while supporting competencies directly relevant to clinical practice.

### Accreditation as a Catalyst for Educational Innovation

Although accreditation standards for audiology education permit flexibility in curricular design, this flexibility has also contributed to uneven adoption of innovative instructional methods across programs. Compared with other health professions with more explicit competency benchmarks, audiology accreditation standards offer limited guidance regarding the scope, frequency, and assessment of simulation-based and clinical problem-solving experiences.

Revising accreditation standards to include clearer expectations for simulation, structured clinical reasoning, and competency thresholds could serve as a catalyst for educational reform. Establishing minimum expectations for simulated cases, SP encounters, and interprofessional learning experiences across the AuD curriculum would promote national consistency while still allowing programs to innovate in how these experiences are delivered. Importantly, such guidance would support measurable outcomes directly linked to workforce readiness and scope-of-practice competencies.

### Investing in Human Capital and Lifelong Learning

Technological innovation has long driven progress within audiology, yet comparable investment in educational innovation and human capital has lagged. While hearing technologies continue to advance rapidly, the profession's capacity to meet expanding clinical and public health demands ultimately depends on the preparation, adaptability, and sustained development of its workforce. Education must therefore be viewed not as a finite process culminating in graduation, but as the foundation for lifelong learning and professional growth.

Preparing audiologists to practice to the full extent of their professional license—including advanced diagnostics, vestibular assessment, and participation in interdisciplinary decision-making—requires educational models that emphasize clinical reasoning, accountability, and confidence. Innovative clinical education, grounded in consistent competencies and supported by accreditation reform, provides the infrastructure necessary to achieve these outcomes.

By intentionally aligning educational strategies with workforce needs, accreditation standards, and professional identity formation, audiology education can move beyond incremental change toward a cohesive and forward-looking model. Such alignment strengthens the profession's capacity to advocate for its role within healthcare, advance scope-of-practice initiatives, and meet the diverse needs of the populations it serves.

### Professional Identity as an Educational and Workforce Outcome

Professional identity in audiology is not formed at a single point in time, nor does it emerge in isolation. Rather, it develops through sustained engagement with the profession's values, expectations, and responsibilities and continues to evolve throughout a clinician's career. Within this framework, professional identity is best understood as an outcome of educational structure, clinical experience, and professional socialization—not merely as an individual or abstract construct.

For audiology, professional identity is inseparable from how clinicians are prepared to function within the broader healthcare system. Educational experiences that emphasize diagnostic reasoning, clinical accountability, and interprofessional collaboration reinforce audiologists' roles as autonomous healthcare providers. Conversely, inconsistent training models and limited opportunities for clinical leadership during education risk perpetuating ambiguity regarding scope of practice and professional standing.

Professional identity formation begins during training and is shaped by both explicit and implicit expectations communicated within educational environments. AuD programs embedded within health sciences or medical school settings often provide early exposure to interdisciplinary care models, enabling students to internalize professional norms aligned with contemporary healthcare practice. These experiences promote confidence, clarify professional boundaries, and normalize audiologists' participation in diagnostic and treatment decision-making alongside other healthcare professionals.

Consistency in educational experiences also plays a critical role in identity formation. When students across programs engage in comparable levels of clinical problem-solving, simulation, and patient communication training, they graduate with a shared understanding of professional competence and responsibility. Accreditation standards that more clearly articulate competency expectations can therefore contribute not only to workforce readiness but also to a unified professional identity across the discipline.

As audiology continues to expand toward its fullest scope of practice, professional identity must evolve in parallel. Preparing graduates to engage confidently in advanced diagnostics, interdisciplinary consultation, and patient-centered care requires educational models that support autonomy, accountability, and lifelong learning. These attributes are reinforced when audiologists view themselves not solely as technology specialists, but as diagnosticians and healthcare providers whose expertise directly influences patient outcomes.

Ultimately, a coherent professional identity strengthens the profession's capacity for advocacy, legislative engagement, and public recognition. When audiologists share a common foundation of educational experiences and competencies, they are better positioned to articulate their value within healthcare systems and to advance the profession collectively. Aligning professional identity formation with educational innovation and accreditation reform ensures that audiology moves forward with a unified voice—one grounded in clinical excellence, consistency, and preparedness to meet evolving public health needs. In this way, professional identity becomes not an abstract outcome, but a measurable product of educational structure, accreditation expectations, and clinical training consistency.

### Aligning Education, Workforce, and Professional Identity

Interprofessional practice represents the most visible test of whether alignment among education, workforce readiness, and professional identity has been achieved. Audiology stands at a pivotal moment in its professional evolution. Increasing demand for services, expanding scopes of practice, and shifting student demographics collectively challenge traditional educational models to do more than sustain the status quo. This manuscript argues that the future vitality of the profession depends on intentional alignment across four interdependent domains: workforce readiness, accreditation reform, consistency of competencies, and professional identity formation.

Workforce readiness begins at the point of entry into the profession. Reliance on passive recruitment pathways and narrow undergraduate pipelines limits both the size and diversity of the audiology workforce. Broadening outreach to students from varied educational backgrounds and life experiences is essential; however, recruitment alone is insufficient. Educational structures must also be intentionally designed to support contemporary learners while preparing graduates to function confidently and competently within increasingly complex healthcare environments.

Central to this preparation is the need for greater consistency in clinical competency development. Traditional reliance on variable clinical rotations has contributed to uneven skill acquisition and inconsistent readiness for independent practice. Integrating structured clinical problem-solving, simulation, and SP experiences into the core curriculum offers a scalable and equitable solution. These approaches shift audiology education from a reactive model to a proactive, competency-based framework that aligns more closely with best practices across other health professions.

Accreditation standards serve as a critical lever for advancing this transformation. While flexibility in curricular design allows for institutional innovation, the absence of clearly articulated expectations for simulation, competency thresholds, and clinical reasoning outcomes has limited progress at a national level. More explicit accreditation guidance would promote consistency across programs while preserving autonomy in instructional delivery. Such reform would not only strengthen educational quality but also reinforce accountability to the workforce and public health needs.

Professional identity emerges at the intersection of these educational and structural elements. When audiologists are trained through consistent, rigorous, and medically aligned educational models, they develop a shared understanding of their role as diagnosticians, healthcare providers, and interdisciplinary collaborators. A unified professional identity strengthens the profession's capacity for advocacy, legislative advancement, and public recognition, positioning audiology as an essential contributor to patient-centered care.

Moving forward, coordinated action across the profession is required. Educators are called to critically examine curricula and clinical training models, prioritizing competency-based outcomes, simulation, and interprofessional learning experiences that reflect contemporary practice demands. Accreditors are encouraged to consider how clearer expectations for clinical reasoning, simulation-based education, and competency thresholds can support national consistency while fostering innovation. Professional organizations play a pivotal role in aligning educational reform with workforce needs by supporting research, disseminating best practices, and advocating for policies that reinforce audiology's evolving scope of practice.

The challenges facing audiology education are not unique; however, the opportunity to address them is timely. By embracing innovation in clinical education, investing in human capital, and aligning accreditation with workforce readiness and professional identity formation, audiology can move forward with clarity and cohesion. Such coordinated efforts offer a path toward a resilient, adaptable, and unified profession—one prepared to meet the evolving demands of healthcare and the diverse populations it serves. The implications of educational reform are perhaps most visible in the context of interprofessional practice.

### Interprofessional Practice

As audiology looks toward the future, one reality is clear: to remain integral within the healthcare system, the profession cannot operate in isolation. Hearing and balance disorders are complex, and as the profession continues to emphasize person-centered care, collaborative practice must remain central to service delivery. Accordingly, audiology education must continue to evolve to prepare future clinicians for effective participation within multidisciplinary healthcare teams.

Audiology has a long history of collaboration with otolaryngology and speech-language pathology; however, the profession must broaden this perspective to more fully engage with medicine, nursing, social work, pharmacy, occupational and physical therapy, mental and behavioral health, and nutrition. Integrated care models ensure that physical, emotional, and psychosocial factors are addressed in holistic treatment planning. Such collaboration improves communication and coordination among providers, reduces fragmented care, accelerates recovery, and fosters patient trust and well-being. 19

As healthcare becomes increasingly specialized, multidisciplinary assessments allow teams of experts to deliver comprehensive care within a single setting, reducing delays and minimizing the burden of multiple appointments. With growing demand for coordinated and personalized care, multidisciplinary clinics are poised to play a central role in the future of healthcare delivery. 20 Audiology education must therefore intentionally model and prepare students to navigate these environments—both in the classroom and through clinical practicum experiences that provide firsthand exposure to team-based care.

Teamwork training should begin early in AuD programs. Medical education offers a useful framework, progressing from implicit team learning—such as problem-based and team-based learning activities—to explicit team training through clinical simulations that include direct instruction and feedback on collaborative performance. 21 As learning advances from foundational knowledge to hands-on clinical practice, educational experiences should increasingly emphasize interprofessional and workplace-based collaboration.

Early training should focus on developing communication competencies, including active listening, clear and concise information exchange, effective questioning, and conflict resolution. Clear expectations regarding roles and responsibilities within teams, coupled with consistent feedback through direct observation, peer input, and self-reflection, are essential. Learning activities should center on realistic case studies and collaborative problem-solving that address complex patient needs. Both intra- and interprofessional experiences are necessary to help students understand collaboration within audiology teams and across healthcare disciplines. Leadership skills can also be cultivated as students learn to guide team efforts while fostering inclusive, respectful professional environments. 22

Although AuD programs are housed within diverse academic structures, intentional efforts must be made to build and sustain relationships with other health professional training programs. Many institutions offer IPE offices that provide infrastructure and expertise to support this work. Clinical practica should also include rotations in settings such as craniofacial teams, developmental disability clinics, traumatic brain injury and concussion programs, and geriatric care teams. Importantly, audiology must not assume that inclusion on these teams will be offered by default. The profession must actively advocate for its expertise in hearing, balance, and communication disorders. When advocacy and collaboration are modeled during training, graduates are better prepared to meet the demands of modern healthcare. The strategic implications extend beyond clinical quality: audiologists who are visibly and routinely integrated into multidisciplinary care teams occupy a professional position in which their services are recognized as medically necessary components of coordinated care—a positioning that directly strengthens the case for Medicare practitioner reclassification, supports the medical necessity determinations that drive reimbursement, and reinforces the scope-of-practice authority that the articles Audiology at the Intersection of Labor, Revenue, and Professional Taxonomy and Audiology in 2050 of this issue" identify as foundational to professional autonomy. IPE, in this light, is not simply good clinical preparation—it is a structural investment in audiology's long-term professional standing within the healthcare system.

## Conclusion

In closing, we reaffirm our commitment to a proactive approach to audiology education—one that elevates current standards by drawing from successful models across healthcare professions and responding to the continued evolution of audiologic scope of practice. Achieving this vision will require AuD programs to critically evaluate existing curricula, remain reflective, and remain open to adopting innovative educational strategies.

We conclude with several guiding questions for educators, accreditors, and professional leaders as the profession looks toward the next 25 years:

- What attributes, educational backgrounds, interpersonal skills, and technical competencies should define future audiology students?
- How are individual AuD programs positioning themselves to prepare graduates for the next generation of audiologic practice?
- Do current accreditation standards continue to align with the profession's long-term vision and evolving healthcare demands?
- How should audiology education further integrate and ensure meaningful interprofessional learning experiences?

Addressing these questions collectively will help ensure that audiology education remains aligned with workforce sustainability, professional identity, and the future needs of the patients and communities it serves.

Educational reform cannot independently resolve the reimbursement constraints, scope limitations, and workforce sustainability challenges documented throughout this special issue. Those require policy action, regulatory modernization, and coordinated professional advocacy. What education can and must do is ensure that the profession's structural reform agenda is not undermined from within—that graduates enter practice fully prepared to exercise the clinical authority being sought, to function as autonomous diagnosticians rather than device-adjacent technicians, to advocate effectively for scope modernization, and to build the professional identity that makes the case for practitioner recognition credible. The analyses presented in this issue are consistent with a profession whose external reform agenda is placed in tension with its internal educational reality when graduates are trained to practice at less than full doctoral scope, when clinical education is financed through structures that impose unsustainable debt, and when accreditation standards codify minimum compliance rather than drive excellence.

Closing that gap—through competency-based curricula, reformed clinical training structures, strengthened accreditation standards, and intentional professional identity formation—is the specific, actionable contribution that audiology education can make to the profession's long-term sustainability. If these educational conditions remain unaddressed, the structural reform agenda faces a persistent internal constraint—one that policy change and regulatory modernization alone are unlikely to overcome, regardless of how much progress is made on the external levers this issue identifies.
